# Emotional pathways to aggression: An experience sampling study of early adolescents

**DOI:** 10.1111/jora.70292

**Published:** 2026-10-15

**Authors:** Shaghayegh Sheri McVay, Jonathan Bruce Santo, Ryan E. Adams, William M. Bukowski

**Affiliations:** ^1^ Department of Developmental Psychology University of Nebraska at Omaha Omaha Nebraska USA; ^2^ Division of Developmental and Behavioral Pediatrics Cincinnati Children's Hospital Medical Center Cincinnati Ohio USA; ^3^ Department of Psychology Concordia University Montréal Québec Canada

**Keywords:** adolescence, adolescents, aggression, emotional dysregulation, emotional reactivity, physical aggression, relational aggression

## Abstract

The high prevalence of aggressive behavior among adolescents represents one of the most pressing challenges facing society today, contributing to significant social, emotional, and academic consequences. The present study used longitudinal data collected via the Experience Sampling Method (ESM) to investigate physical and relational aggression among a sample of Canadian adolescents (*M*_age_ = 10.85 years; 54% male). Through frequent assessments during the school day and the integration of self‐report and peer‐nomination data, this design enabled examination of fluctuations in emotional reactivity, emotional dysregulation, and aggressive behavior. Additionally, the study tested the potential indirect effect of emotional reactivity in the association between emotional dysregulation and physical and relational aggression. The results of multilevel modeling revealed that boys were rated higher on emotional dysregulation and physical aggression, whereas girls were more likely to be nominated for relational aggression. Emotional dysregulation was positively related to physical and relational aggression, but gender moderated this effect: emotional dysregulation predicted aggressive behavior more strongly among boys. Emotional reactivity to negative experiences also showed gender‐specific associations, such that greater emotional reactivity was associated with greater physical aggression only among boys. Finally, among boys, the association between emotional dysregulation and physical aggression was partially attributed to the potential indirect effect of emotional reactivity, underscoring the role of real‐time emotional processes in shaping aggressive behavior. The findings highlight the importance of examining in situ emotional reactions and their relation to aggressive behavior. Implications for intervention and future research, particularly regarding cultural generalizability, are discussed.

## INTRODUCTION

As a pervasive issue worldwide, adolescent aggression remains a serious public health concern (Pellegrini et al., 1999; Vega et al., 2022) that, at its worst, is associated with interpersonal, community, and school violence (Ioannou et al., 2015; Liu, 2004; Wike & Fraser, 2009). Even in less extreme cases, aggression is a substantial risk factor for peer difficulties (e.g., Bass et al., 2022) and long‐term maladjustment. Aggression is also a common reason young individuals are referred to mental health professionals (Scott et al., 2018) and can manifest as a symptom in various psychological conditions including depression (Roland, 2002), post‐traumatic stress disorder (Taft et al., 2017), bipolar disorder, attention deficit hyperactivity disorder (Doerfler et al., 2011), and conduct disorder (Buitelaar et al., 2013). As a personality trait, aggressive tendencies often emerge early in development and show moderate to high stability over time (Adams et al., 2005; Olweus, 1979; Scott et al., 2018). Among individuals with high levels of aggression, behaviors typically follow a steady, linear increase without proper intervention (Liu, 2004; Scott et al., 2018). Given the early emergence, stability, and wide‐ranging consequences of aggressive behavior, this study aims to assess underlying mechanisms that drive aggression, particularly emotional processes including dysregulation and reactivity.

Although a universally agreed‐upon definition of this construct does not exist, aggression is broadly characterized as behaviors maliciously intended to harm another person (Bukowski & Abecassis, 2007; Gendreau & Archer, 2005). Beyond predicting later misconduct, aggressiveness is also associated with diverse patterns of social maladjustment, including increased risks of teenage pregnancy and criminal arrests (Cairns & Cairns, 1994; Ladd, 2022). The United Nations Educational, Scientific, and Cultural Organization (UNESCO, 2018) estimates that one‐third of students between the ages of 9 and 15 have engaged in a fight, and 32% have been beaten within the past year, underscoring the worldwide scope of this problem.

Given the significant implications of aggressive behavior, developing a clear understanding of the processes that drive aggression during adolescence is a critical step toward effective prevention and intervention. The challenge is that aggressive behavior often emerges in response to immediate social experiences and emotional reactions. However, much of the existing research relies on cross‐sectional or widely spaced longitudinal designs that assess aggression retrospectively. Such approaches fail to capture the immediate emotional responses and situational triggers that precede aggressive behavior. As a result, it remains difficult to determine how adolescents' emotional tendencies (e.g., emotional dysregulation) interact with real‐time social experiences to produce aggressive behavior in the moment. In contrast, intensive longitudinal approaches, such as the Experience Sampling Method (ESM), offer a unique opportunity to address these limitations by repeatedly assessing adolescents' experiences and emotions in naturalistic settings. This methodology also allows researchers to examine how negative peer interactions are associated with immediate emotional responses. Therefore, in the present study, frequent assessments across multiple school days (five times per day) and the combination of self‐reports and peer nominations enable the capture of fluctuations in adolescents' emotional reactivity, emotional dysregulation, and aggressive behaviors.

### Understanding contextual dynamics in adolescent aggression

Consistent with Bronfenbrenner's (1979) ecological paradigm, development does not occur in a vacuum; thus, understanding aggression necessitates a contextual perspective that considers the social environment in which it occurs (Bass et al., 2018; Bukowski & Abecassis, 2007). Similarly, although adolescent aggression is frequently associated with social skill deficits or behavioral problems, it is important to acknowledge that development is influenced by a wide range of social and environmental factors (da Cunha et al., 2021). Additionally, research has demonstrated the important role of emotion in motivating aggression (for a review, see Roberton et al., 2012), and it is clearly not as simple as the traditional frustration‐aggression hypothesis portrays (Miller, 1941), which points to the notion that through experience, individuals learn to respond to frustration with aggressive behavior (Bushman & Huesmann, 2010). Rather, there is likely to be a complex interplay of intraindividual variability related to emotion processes. In fact, the General Aggression Model (GAM; Anderson & Bushman, 2002) suggests that an individual's decision to engage in aggression is influenced not only by situational factors but also by individual factors, such as emotionality (Nelson et al., 2022).

Similarly, according to the contextual‐developmental perspective (Chen, 2011), peers represent a key context in which social interactions play an important role in socioemotional development. Drawing on Vygotsky and Cole (1978) sociocultural theory and Bronfenbrenner's (1979) socioecological framework, the contextual‐developmental perspective suggests that self‐control shapes social evaluations and reactions during peer interactions, thereby influencing and regulating social behaviors (Chen et al., 2022). In this context, self‐control denotes the ability to manage emotional impulses, which is fundamental for fostering stable and adaptive social relationships (Chen et al., 2022). When regulatory capacities are weak, adolescents may be more likely to respond to challenging situations with heightened emotional reactions and difficulty managing impulses, which can increase the likelihood of aggressive responses. Thus, emotional dysregulation and emotional reactivity may represent key mechanisms through which peer contexts contribute to both physical and relational aggression.

### Types of aggression and gender differences

Aggression encompasses a wide variety of behaviors that vary in their underlying motivations and outward expressions (Nelson et al., 2022). These variations matter because different forms of aggression are expressed by different individuals, triggered in different situations, and have different implications and outcomes. This underscores the importance of distinguishing between types of aggressive behavior to disentangle the processes and mechanisms of aggression. In this regard, physical and relational aggression follow different developmental trajectories and have distinct consequences (Nelson et al., 2022). According to Bushman and Huesmann (2010), physical aggression involves physical force or threat to hurt someone (e.g., hitting, kicking, shooting) or to take away something (i.e., deprivation). In contrast, relational or social aggression involves intentionally targeting an individual's social status or feelings of acceptance, often through behaviors such as spreading rumors, gossiping, or social exclusion (Bass et al., 2018; Prinstein et al., 2001).

The distinction between physical and relational aggression is also important due to gender differences in expression and development of aggressive tendencies. A substantial body of literature has demonstrated that there are gender differences in the normativeness of each type of aggression (Busching & Krahé, 2015). Although there are exceptions, many studies have found that boys tend to exhibit more physical aggression than girls (Archer, 2004; Lansford et al., 2012; Ostrov & Crick, 2007). In contrast, girls often demonstrate a proclivity for relational aggression (Bass et al., 2022; Ostrov & Crick, 2007), which may increase during early to mid‐adolescence (Bowie, 2007). These differences may be in part due to gender‐typed socialization and reinforcement that may explain gender differences in emotional processes related to aggression. For example, boys are more likely to be rewarded for assertive, dominant, and aggressive expressions and may be given encouragement for expressing anger (Velásquez et al., 2010). On the other hand, girls are generally guided to suppress such emotions to build relationships instead. In fact, many girls, including those who are well‐adjusted, seem to engage in relational aggression from time to time as a normalized pattern of behavior within social relationships and in response to conflicts (Bowie, 2007), suggesting that the behavior itself may be a learned response for coping with social emotions.

Furthermore, for boys, a tendency toward physical aggression may be uniquely related to the experience and regulation of anger (Berkowitz, 1990; Eisenberg et al., 2001). Alternatively, meta‐analytic research has also identified that gender differences in thresholds of emotional arousal may influence apparent gender differences in aggressive responses, such that males have more difficulty modulating behavioral responses to emotionally evocative situations (Knight et al., 2002). Multiple studies have also shown that the association between negative life experiences and later aggression can be explained by emotional dysregulation, with some finding this to be the case only for males and not for females (e.g., Cooley & Fite, 2016; Hay, 2007; Röll et al., 2012).

### The role of emotions in aggressive behaviors

Emotions shape the flow and outcome of interpersonal interactions (von Salisch et al., 2022). These multicomponent processes (Gross, 2002) arise in response to events or situations that are personally meaningful to an individual, and a key aspect of optimal development is learning to manage and control them in appropriate and adaptive ways (Morris et al., 2007). In this respect, emotion regulation refers to the processes through which individuals manage the emotions they experience, their timing, and how they are expressed (Gross, 2002). This capacity plays a central role in both internalizing and externalizing outcomes, as effective regulation helps prevent overwhelming negative emotions and reduces the likelihood of maladaptive behavior (Kim & Cicchetti, 2010). Emotion dysregulation, by contrast, reflects maladaptive responses to emotional states (Werner & Gross, 2010) and is considered a pathway through which negative affect influences the development of psychopathology (Donahue et al., 2014). In addition, emotional dysregulation has been shown to be related to aggression and the expression of aggressive behavior (Cohn et al., 2010; Roberton et al., 2012). Complementing these perspectives, emotional reactivity involves the tendency to react to situations in a specific way and is usually indexed by intensity, duration, and latency (Gartstein et al., 2016). Although emotional reactivity and other emotional responses may be influenced by neurophysiology and temperament, they are also context‐dependent, and situational factors can substantially influence how these tendencies are expressed and the extent to which individuals are vulnerable to developing internalizing or externalizing behaviors such as aggression and psychopathology (Silva et al., 2018; Zimmermann & Iwanski, 2018). Importantly, adolescents may vary in the extent to which negative peer experiences evoke emotional responses. Ultimately, the consensus is that emotions are products of relationships with others (Cole et al., 2004; Morris et al., 2007), and others' behaviors constitute antecedents for emotions (von Salisch et al., 2022). Consequently, adolescence represents a critical period for examining the relationship between emotional responses and externalizing behaviors, such as aggression. This stage is a formative period in the development of emotion regulation and emotional reactivity, as individuals experience substantial biological, psychological, and social changes (Bookhout et al., 2018; Zimmermann & Iwanski, 2018).

However, although self‐regulation has been recognized as an important element in inhibiting aggressive behavior (Bushman & Huesmann, 2010), the interplay between situationally triggered emotions and individual differences in emotional processing remains understudied. In particular, the literature lacks clarity on the processes by which emotional responses to negative experiences in the moment affect outcomes, including aggressive behavior (Hubbard & Dearing, 2004). One possible explanation could be that adolescents who struggle with emotional regulation may react more strongly to negative peer experiences, which in turn increases the likelihood of aggressive behavior.

### Experience sampling of emotions

According to the General Aggression Model, person and situational factors influence feelings, arousal, and thoughts (Allen et al., 2018). In this context, person factors refer to between‐individual variability in how people respond to different situations, such as emotionality and personality (Nelson et al., 2022). Situation factors, by contrast, are external triggers that may act as a cue to trigger aggression, such as provocation, social rejection, stress, frustration, or even an aggressive cue, like a weapon (Nelson et al., 2022). These factors interact to shape arousal and cognition, which in turn affect the likelihood of the expression of aggressive behavior (Allen et al., 2018). Therefore, understanding the mechanisms underlying behavior requires research designs that are attuned to dynamic, real‐time processes. In this regard, intensive, short‐term longitudinal research involving multiple repeated measurements via the experience‐sampling method is ideal for understanding how social and emotional processes unfold in daily life. Although there is variability in how experience sampling is conducted, these methods often involve one or more data collections per day for a period of a few days to up to one month (Csikszentmihalyi & Larson, 2014). In addition, in contrast to traditional approaches, experience sampling allows for investigation of time‐dependent emotion traits, which are critical to understanding how emotion influences daily behavior (Morrow et al., 2014).

### The current study

The goal of the current study was to fill key gaps in understanding how emotions and daily experiences relate to aggressive behavior. Specifically, this study examined the links between negative peer experiences, emotional reactivity, and individual differences in emotional dysregulation in relation to both physical and relational aggression, as well as gender differences in these associations, using a sample of Canadian adolescents from a public school in Montreal. Based on these objectives, multilevel structural equation modeling was utilized to test the following hypotheses:

Hypothesis #1: At the within‐individual level, negative peer experiences (in the moment) will be positively related to negative emotions. To explain, when adolescents experience negative peer interactions, they are more likely to feel negative emotions.

Hypothesis #2: There will be significant variability between adolescents in the effect above (labeled emotional reactivity).

Hypothesis #3: At the between‐individual level, peer nominations of emotional dysregulation will be positively related to peer nominations of both physical and relational aggression.

In other words, adolescents who are nominated by peers as having trouble managing their emotions are more likely to be nominated for both physical and relational aggression.

Hypothesis #4: Emotional reactivity will act as a potential indirect pathway in the association between emotional dysregulation and both physical and relational aggression.

Hypothesis #5: The indirect association between emotional dysregulation and aggression through emotional reactivity will be significantly stronger for boys than for girls. In other words, the pathway from emotional dysregulation to aggression through emotional reactivity is expected to be more pronounced for boys.

## METHOD

### Participants

This study involved a secondary analysis of anonymized data from 109 early adolescents in 5th and 6th grades (*M*_age_ = 10.85 years; 54% male). Participants were recruited from a public school located in the Montreal metropolitan area, Canada. Recruitment occurred at the classroom level, such that all students within participating classrooms were invited to participate. Inclusion criteria required that students return signed parental consent forms and provide assent themselves. The population was primarily middle‐class and European‐Canadian, encompassing both English‐ and French‐speaking neighborhoods. Although English was not listed as the first language for some participants, they were all fluent in English, and school instruction was entirely in English. Prior to data collection, letters describing the study procedures and participants' rights were sent to parents. The return rate for consent forms was 85%, resulting in a final participation rate of 82%. Only students who obtained parental consent and provided their own assent participated in the study.

We also report that this research was not preregistered, and no data were excluded. In addition, consistent with the guidelines of Hox and Maas (2001), the sample size was deemed sufficient given the complexity of the model. It is also important to note that the data used in the present study were drawn from a broader longitudinal experience sampling project examining peer victimization and depressive symptoms. Extending prior work using this dataset, the present study addresses a distinct developmental question by examining emotional reactivity and emotional dysregulation as potential pathways underlying physical and relational aggression during early adolescence.

### Procedures

Longitudinal data collection was completed over four consecutive school days (Tuesday through Friday). Prior to the first day of longitudinal data collection, participants completed a series of self‐report and peer‐report measures, including sociometric ratings and peer nominations of socially relevant characteristics and behaviors. Experience sampling assessments were completed five times per day for each of the four days. During each assessment, participants were asked to report about their experiences from 20 min prior, including what they were doing and several ratings characterizing that experience and their feelings at the time. These assessments occurred approximately 30 min after waking (*M* = 6:45 A.M., *SD* = 25.80 min), after the start of school (*M* = 8:15 A.M., *SD* = 24.60 min), after first recess was over (*M* = 10:00 A.M., *SD* = 29.40 min), right after lunch (*M* = 12:25 P.M., *SD* = 21.00 min), and at the end of the school day (*M* = 2:00 P.M., *SD* = 16.20 min). To minimize participant burden, assessments were intentionally brief and scheduled during natural transitions in the school day (e.g., after recess or lunch). The experience sampling protocol focused on adolescents' immediate experiences and emotions and was designed to be minimally disruptive to regular classroom activities. Furthermore, data collection occurred over only four consecutive school days to reduce participant fatigue while still capturing fluctuations in daily emotional and social experiences.

In addition, experience sampling assessments were completed using paper‐and‐pencil booklets administered at each assessment period. Researchers were present at the school throughout the data collection period and provided prompts and reminders to students at each scheduled assessment period to ensure the timely completion of the experience sampling measures. With the exception of the first daily assessment completed at home approximately 30 min. after waking, all experience sampling assessments were completed during school hours in supervised classroom settings. Compliance was monitored by trained research staff, who administered the assessments, provided instructions and reminders, and directly collected completed assessment booklets at each time point. Across the four‐day protocol, participants could complete up to 20 experience sampling assessments (five per day over four days), yielding 2180 possible assessments across participants. The present analyses included 1965 completed experience sampling assessments, corresponding to an overall completion rate of approximately 90.14%. Missing data were handled using full information maximum likelihood.

### Measures

#### Experience sampling assessments

At each assessment (five assessments per day for four days), participants were asked to report their experiences with their peers from 20 min prior. For each recorded experience, participants were asked, “How was the interaction with your friends/classmates?” and “How do you feel about it?” The valence of each experience was on a 7‐point scale ranging from 1 (very *positive*) to 7 (*very negative*). These ratings were used to create the observed predictor variable, negative peer experiences (Cronbach's alpha = .72).

#### Emotional reactivity

In addition, during each assessment, participants were asked to report their emotions at that time. The relationship between peer experiences and emotional responses served as an index of emotional reactivity. To elaborate, emotional reactivity was operationalized as adolescents' momentary negative emotional responses to daily peer experiences and was estimated using repeated self‐reports collected through the experience sampling method. Thus, emotional reactivity reflected a within‐individual process capturing fluctuations in affective responses across situations.

Negative emotional responses to each experience were assessed using the following five self‐report items: “I have felt frustrated,” “I have felt angry or hostile,” “I have felt unhappy,” “I have felt upset because something happened that I did not expect,” and “I have found it hard to concentrate on what's happening” (*ω* = 0.80). These items served as indicators of the latent factor negative emotions, which was modeled as the Level 1 outcome. All items were answered on a scale of 1 (*not at all*) to 5 (*very much*).

#### Emotional dysregulation, physical and relational aggression

Emotional dysregulation and physical and relational aggression were measured using peer nominations. To assess these constructs, participants completed an unlimited‐choice peer‐assessment questionnaire adapted from the Revised Class Play (RCP; Masten et al., 1985). They were provided with a list of all participating classmates and a series of descriptions of characteristics or behaviors. For each description, participants were asked to select the classmates who fit those characteristics or behaviors.

To minimize potential bias, peer nominations were completed confidentially, and participants were instructed to respond independently based on their observations of classmates' typical behaviors. In addition, the unlimited‐choice nomination procedure reduced pressures associated with forced‐choice formats and allowed participants to nominate peers more naturally. Peer‐nomination approaches have been broadly used in developmental research because peers are often well positioned to observe socially salient behaviors, including aggression and emotional dysregulation, in everyday peer interactions. Moreover, peer nominations may reduce some limitations associated with self‐report measures of aggression, particularly social desirability biases.

Given the age of the sample, only same‐gender nominations were used, and the peer‐nomination counts were not standardized based on the size of the same‐gender nominator pool within each classroom. Classroom sizes ranged from 16 to 27 students, and the proportion of boys across classrooms ranged from 40.90% to 68.20%. Thus, differences in classroom size and gender composition resulted in some variation in the number of same‐gender peers available to provide nominations. We therefore tested whether variation in peer‐group size was associated with the number of nominations received. These analyses indicated that peer‐group size accounted for less than 1% of the variability in nomination scores, suggesting that differences in nomination opportunities contributed minimally to the observed variation in peer nominations. Given this negligible association, we retained the raw nomination counts in the analyses. Nevertheless, differences in the size and gender composition of the nomination pools should be considered when interpreting the results.

Emotional dysregulation was assessed with four items: “someone who is cranky,” “someone who has trouble controlling themselves,” “someone who is easily annoyed,” and “someone who reacts quickly without thinking” (*ω* = 0.84). Meanwhile, physical aggression was assessed with three items: “someone who hurts others physically,” “someone who hits, pushes, or shoves people,” and “someone who gets involved in physical fights” (*ω* = 0.88). Finally, relational aggression was assessed with three items: “someone who tries to keep others out of the group when it's time to play,” “someone who talks bad about others behind their backs to hurt them,” and “someone who when mad at someone, ignores or stops talking to him/her” (*ω* = 0.76).

### Statistical analyses

We used a multilevel structural equation modeling (MSEM) approach to account for both within‐individual fluctuations across days and time points and for between‐individual indirect effects (Muthén & Muthén, 2006). The analyses were conducted in M‐plus, version 8.11 (Muthén & Muthén, 1998–2017) using the robust maximum likelihood (MLR) estimator. Missing data were also addressed using full information maximum likelihood (FIML). At the within‐individual level, the negative emotional response items assessed during the experience sampling procedure were modeled as indicators of a latent factor of negative emotions. Negative peer experiences were then treated as an observed predictor of this latent construct. We then allowed the effect to be random to test for differences (i.e., by creating a latent variable of the between‐individual differences in the association between negative peer experiences and negative emotions). To elaborate, the latent variable of emotional reactivity reflected between‐individual differences in the association between negative peer experiences and negative emotions. Thus, the association was assessed at level 1, and the variability in the effect was assessed at level 2.

At the between‐individual level, peer nominations of emotional dysregulation, physical aggression, and relational aggression were each modeled as latent constructs using their respective nomination items as indicators. Emotional dysregulation was modeled as a predictor of both physical and relational aggression. Moreover, the latent factor of emotional reactivity was used as a potential mediator between emotional dysregulation and both physical and relational aggression. The observed gender variable was subsequently included as a moderator of these associations. Figure 1 depicts these associations. In addition, it is important to mention that separate confirmatory factor analyses were not conducted prior to the MSEM analyses. Instead, the latent constructs were modeled directly within the multilevel structural equation framework, allowing the measurement and structural components to be estimated simultaneously while accounting for both within‐ and between‐individual variability.

**FIGURE 1:**
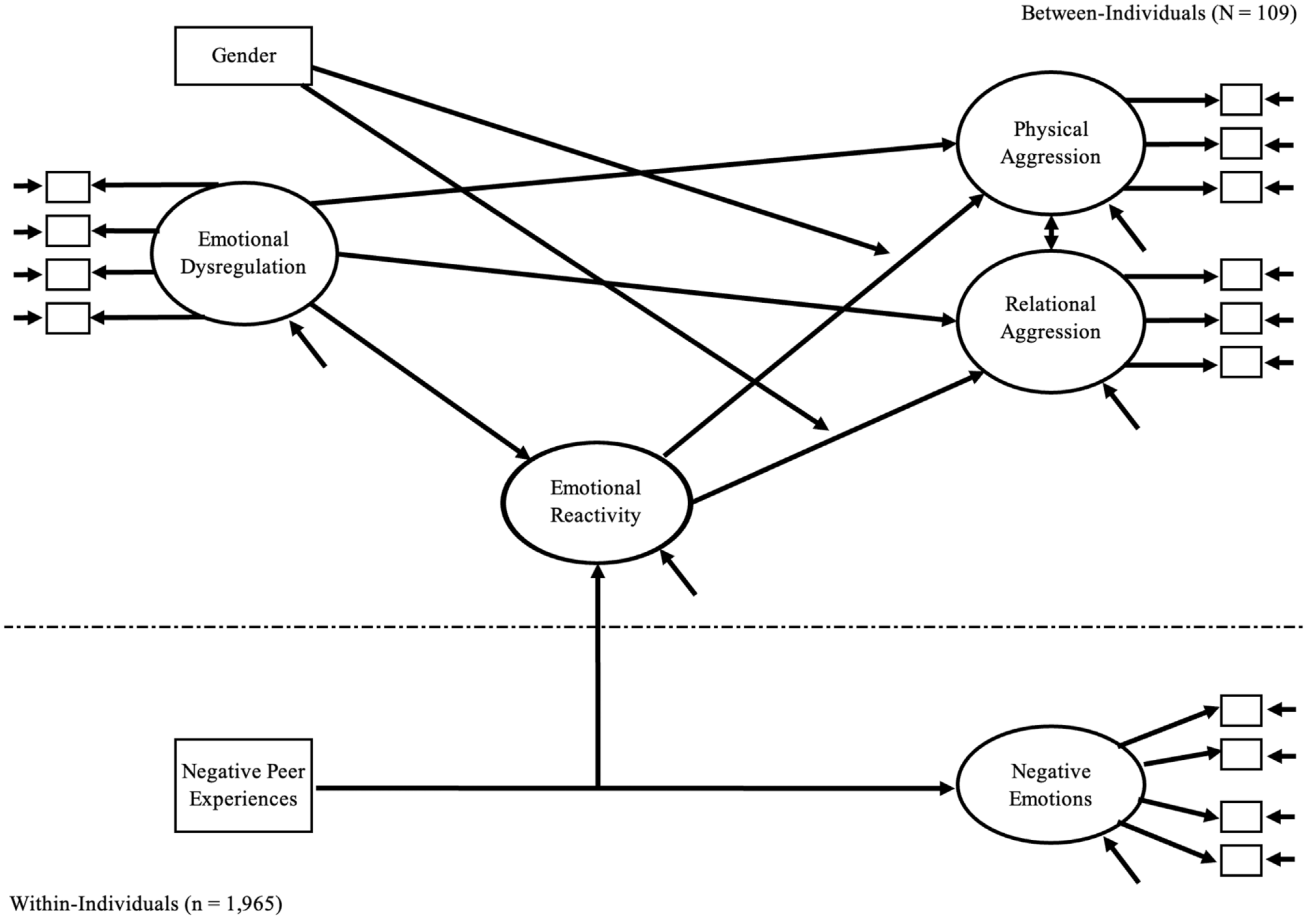
The hypothesized gender‐moderated mediation of emotional reactivity in the association between emotional dysregulation and both physical and relational aggression.

## RESULTS

Tables 1 and 2 present descriptive statistics and correlations for the observed indicators underlying the latent constructs. Although the primary analyses were conducted at the latent‐variable level within the MSEM framework, presenting item‐level statistics enables evaluation of the observed associations among the indicators used to estimate each construct. Accordingly, these correlations should be interpreted as reflecting the relations among the measured items themselves. Furthermore, independent samples *t*‐tests were used to test for potential gender differences (see also Tables 1 and 2). Ultimately, there was a strong correlation between emotions of frustration, anger, and unhappiness, suggesting a coherent negative affect as a result of negative experiences with peers. Aggressive behaviors also showed strong interrelations, particularly among physical aggression items. Aggression was also moderately associated with emotional dysregulation. As expected, boys were rated significantly higher on emotional dysregulation and physical aggression, whereas girls were more likely to be nominated for relational aggression. Figure 2 depicts these differences. However, there were no differences in the experience sampling measures of negative peer experience and/or emotional reactivity (again, Figure 2).

**TABLE 1:** Descriptive statistics and zero‐order correlations for the experience sampling data.

	Full sample		Zero‐order correlations							Boys		Girls		*t* ^*p*^	df
	*M*	*SD*	01.	02.	03.	04.	05.	06.	07.	*M*	*SD*	*M*	*SD*		
01. How was the interaction with your friends/classmate(s)?	3.29	1.94	–							3.40	1.98	3.16	1.88	2.73*	1916.00
02. How did you feel about it?	4.35	1.25	.62*	–						4.41	1.32	4.29	1.17	2.13*	1954.00
03. I have found it hard to concentrate on what's happening	1.98	1.64	−.04	−.03	–					1.83	1.61	2.14	1.67	−4.20*	1942.01
04. I felt upset because something happened that I did not expect	1.95	1.67	.06*	.02	.59*	–				1.85	1.66	2.06	1.68	−2.88*	1955.76
05. I felt frustrated	1.49	1.02	.10*	.09*	.35*	.43*	–			1.52	1.10	1.45	0.93	1.37	1990.77
06. I felt angry/hostile	1.39	.94	.12*	.11*	.30*	.38*	.81*	–		1.41	0.98	1.36	0.89	1.06	1990.52
07. I felt unhappy	1.45	.99	.09*	.08*	.31*	.35*	.71*	.77*	–	1.47	1.04	1.42	0.93	1.23	1989.93

*Note*: Tests for mean differences based on gender are also shown above. For items 01 and 02, participants rated how positive or negative each experience was using a 7‐point scale ranging from 1 (*very positive*) to 7 (*very negative*). For items 03–07, responses were rated on a 5‐point scale ranging from 1 (*not at all*) to 5 (*very much*).

* *p* < .05.

**TABLE 2:** Means, standard deviations and zero‐order correlations for the between‐individual data.

	Full sample		Zero‐order correlations										Boys		Girls		*t* ^*p*^	df
	*M*	*SD*	01.	02.	03.	04.	05.	06.	07.	08.	09.	10.	*M*	*SD*	*M*	*SD*		
01. Someone who hurts others physically	1.03	1.46	–										1.48	1.74	0.50	0.78	3.94*	86.03
02. Someone who hits, pushes, or shoves people	1.05	1.75	.79*	–									1.46	2.21	0.58	0.75	2.93*	75.68
03. Someone who gets involved in physical fights	1.18	2.07	.80*	.78*	–								1.87	2.58	0.37	0.60	4.42*	67.42
04. Someone cranky	1.52	1.67	.40*	.50*	.47*	–							1.84	1.96	1.15	1.16	2.29*	99.71
05. Someone who has trouble controlling themselves	1.61	2.01	.60*	.67*	.70*	.64*	–						1.98	2.31	1.17	1.49	2.24*	103.76
06. Someone who is easily annoyed	1.96	1.71	.21*	.38*	.29*	.54*	.55*	–					1.90	1.80	2.02	1.63	−0.36	111.00
07. Someone who reacts quickly without thinking	1.60	1.70	.60*	.68*	.73*	.54*	.69*	.48*	–				1.89	2.13	1.27	0.91	2.05*	83.92
08. Someone who tries to keep others out of the group when it's time to play	1.69	1.78	.24*	.21*	.31*	.25*	.29*	.17	.30*	–			1.33	1.86	2.12	1.59	−2.40*	111.00
09. Someone who talks badly about others behind their backs to hurt them	1.74	1.63	.20*	.20*	.21*	.23*	.30*	.18	.28*	.70*	–		1.48	1.47	2.06	1.76	−1.92	111.00
10. Someone who, when mad at someone, ignores or stops talking to him/her	1.65	1.60	−.12	−.08	−.11	.17	.10	.48*	.03	.28*	.35*	–	0.98	1.41	2.42	1.47	−5.28*	106.48

*Note*: Range 0–11 (peer nominations) for items 01–03, range 0–10 (peer nominations) for items 04–07, and range 0–9 (peer nominations) for items 08–10.

* *p* < .05.

**FIGURE 2:**
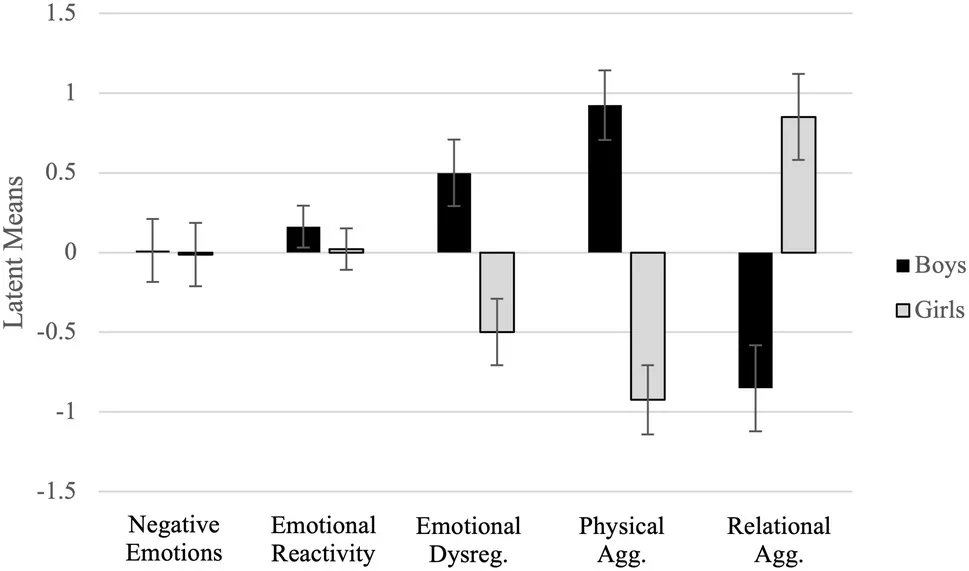
Mean differences as a function of gender (error bars reflect the 95% confidence intervals). Error bars reflect the 95% confidence intervals.

To begin testing the hypotheses, an unconditional model was created with only the negative emotions variable. This model revealed that 45.77% to 60.54% of the variability was at the higher level of the model (i.e., between‐individuals), justifying the multilevel approach. Next, the latent factor of emotional reactivity was created using the negative emotional response items as indicators. Adding this variable reflected a significant improvement to the model (ΔBIC = −347.17). We also modeled the effect of negative peer experiences on the latent negative emotions variable. Consistent with our directional hypotheses and prior literature, one‐tailed significance tests were used for theoretically directional effects. As expected, more negative experiences in the moment were related to more negative emotional responses (*b* = .07, *z* = 1.84, *p*_(1‐tailed)_ < .05). Again, this led to a significant improvement in the model (ΔBIC = −510.13). Thus, hypothesis #1 that at the within‐individual level, negative peer experiences (in the moment) would be positively related to negative emotions was supported. We then modeled the between‐individual variability in the effect of negative peer experiences on negative emotional responses as a latent factor (labeled emotional reactivity moving forward; variance = .25, *z* = 1.91, *p*_(1‐tailed)_ < .05). The prediction that there would be significant variability between adolescents in this emotional reactivity was marginally supported.

Moreover, allowing the effect of negative peer experiences to be random (i.e., assumed to differ between adolescents) significantly improved the model (ΔBIC = −411.13) and reduced the prediction error in the dependent variable, negative emotions, by 28.76%. In other words, the model markedly improved, and the prediction error of the estimation of negative emotions was reduced once we allowed the degree to which negative emotions were impacted by negative peer experiences to vary from person to person. Figure 3 depicts the association between negative peer experiences and emotions along with the variability in emotional reactivity.

**FIGURE 3:**
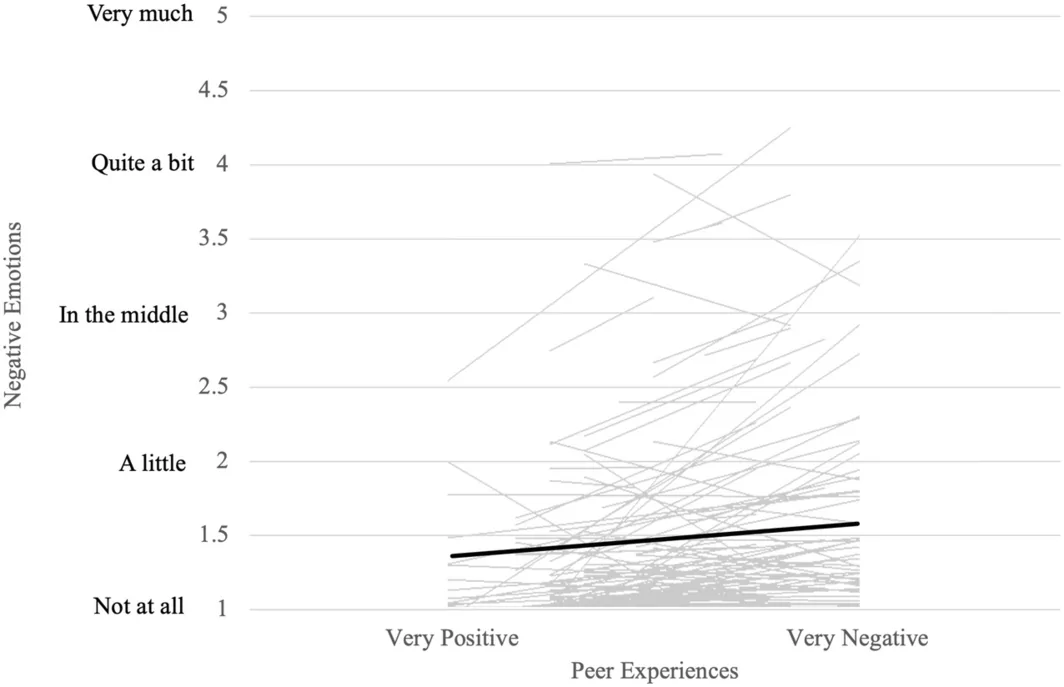
The association between negative peer experiences and emotions. Grayed lines reflect the individual slopes in emotional reactivity.

Next, analyses were elevated to the between‐individual level by modeling the latent factors of both physical and relational aggression and the latent construct of emotional dysregulation as a predictor. Both forms of aggression were weakly positively related (covariance = .49, *z* = 1.85, *p*_(1‐tailed)_ < .05). As expected, more emotional dysregulation nominations were related to greater nominations for physical aggression (*b* = 1.23, *z* = 3.69, *p* < .05, *R* ^2^ = 68.82%) and relational aggression (*b* = .64, *z* = 2.55, *p* < .05, *R* ^2^ = 16.65%), significantly improving the model (ΔBIC = −80.90). As such, hypothesis #3 that emotional dysregulation would be positively related to physical aggression and relational aggression was also supported. It is worth noting that we included the effect of age on emotional dysregulation as a covariate due to a modest association (*b* = .21, *z* = 1.88, *p*_(1‐tailed)_ < .05, Δ*R* ^2^ = 4.83%). Simply, older participants were slightly more likely to be nominated by their peers for the items of emotional dysregulation.

At this point, we tested the potential indirect effect of emotional reactivity in the associations between emotional dysregulation and both relational and physical aggression by folding in the within‐individual latent factor of emotional reactivity using the between‐individual variability in this effect as an additional predictor of aggression. In the resulting model, emotional dysregulation was not related to emotional reactivity, nor was emotional reactivity related to either measure of aggressive behavior. Given these non‐significant effects, unsurprisingly there was no indirect effect of emotional reactivity on the association between emotional dysregulation and physical or relational aggression.

However, in a subsequent model, we included gender. A number of significant effects emerged. Not only were girls significantly lower in emotional dysregulation (*b* = −.47, *z* = −2.06, *p* < .05, Δ*R* ^2^ = 4.09%), but they were lower in physical aggression as well (*b* = −.51, *z* = −2.99, *p* < .05, Δ*R* ^2^ = 12.09%). On the other hand, girls were nominated significantly more for relational aggression (*b* = 1.07, *z* = 4.05, *p* < .05, Δ*R* ^2^ = 4.70%), whereas there was no significant main effect of gender on emotional reactivity. The resulting model was a significant improvement (ΔBIC = −5586.72). It is also important to note that an indirect effect did emerge with the inclusion of gender. Namely, a portion of the effect of gender differences in both physical and relational aggression was indirectly through emotional dysregulation. To explain, part of the reason that boys were more likely to be nominated for physical aggressive behavior was that they were also more nominated for emotional dysregulation, and that was likewise associated with more aggression nominations (Δ*b* = −.41, 90% CI [−.77, −.05], *z*_(1tailed)_ = −1.88, *p* < .05) and similarly among girls for relational aggression (Δ*b* = −.28, 90% CI [−.53, −.03], *z*_(1tailed)_ = −1.84, *p* < .05).

In the final model, we added gender interactions to the associations detailed above. The interactions further elucidated the associations between emotional dysregulation and physical aggression and the indirect effect of emotional reactivity, though not for relational aggression. First, gender significantly moderated the effect of emotional dysregulation on aggression (see Figure 4, top panel). Namely, emotional dysregulation was only positively related to physical aggression among boys as opposed to girls. In addition, gender also significantly moderated the effect of emotional reactivity on physical aggression (see Figure 4, bottom panel). Specifically, greater emotional reactivity to negative peer experiences was associated with greater physical aggression only among boys. Adding these interactions further reduced prediction error in physical aggression by 78.27%. Moreover, this revealed another indirect effect whereby the association between emotional dysregulation and physical aggression was partially accounted for by emotional reactivity (Δ*b* = −2.82, 90% CI [−5.62, −.02], *z* = −1.65, *p*_(1‐tailed)_ < .05), which was stronger for boys than for girls. To explain, in the model with the gender interaction on the association between emotional dysregulation and physical aggression, part of the association was explained by emotional reactivity (further reducing the effect; Δβ = ‐46.01%). As such, the direct association between the two variables (emotional dysregulation and physical aggression) was significantly weaker once the gender interaction was introduced through the emotional dysregulation pathway.

**FIGURE 4:**
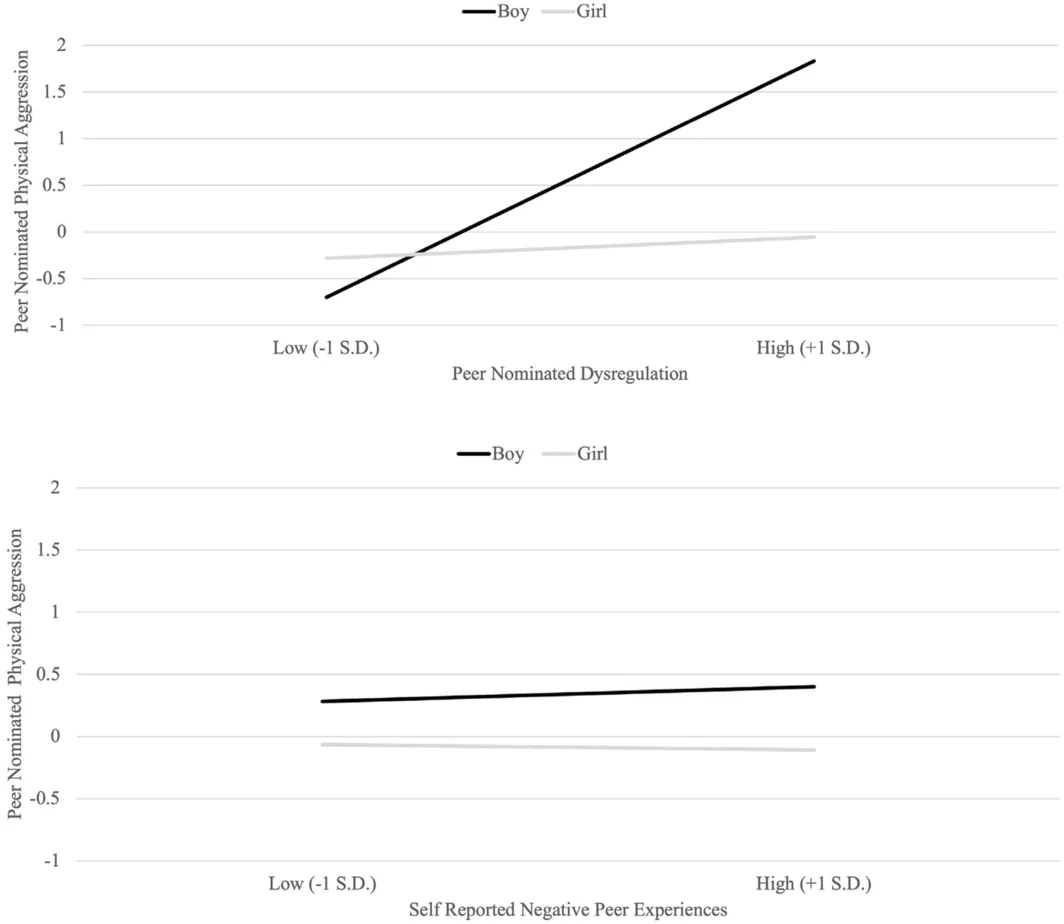
The moderating role of gender. Gender moderates the association between peer nominations of emotional dysregulation and physical aggression (top panel) and the association between the effect of negative peer experiences on peer nominations of physical aggression (bottom panel).

Additional analyses indicated that the association between emotional dysregulation and physical aggression was significant among boys (*b* = 1.13, *z* = 3.29, *p* < .05), but not among girls (*b* = .14, *z* = .84, *p* > .05), providing additional support for the interpretation that this indirect pathway was primarily driven by the pattern among boys. Adding gender interactions significantly improved the model further (ΔBIC = −18.34). As such, hypothesis #4, that the association above would be indirectly accounted for by individual differences in emotional reactivity, *was* supported, but only among boys and only for physical aggression. For boys, emotional dysregulation was tied to more physical aggression partially as a result of the effect of higher emotional reactivity. Girls, however, were less likely to be nominated for emotional dysregulation and, though they were more relationally aggressive, there appeared to be no indirect effect of emotional reactivity in the association. As such, hypothesis 5 was conditionally supported.

## DISCUSSION

The results of this study offer a unique understanding of the emotion‐based elicitation of aggressive behavior, especially among early adolescent boys. This pathway, which involves both trait‐based and situational responses, supports that there may be unique mechanisms underlying common expressions of externalizing behavior for boys. Specifically, the experience sampling method demonstrated that the acute experience of negative peer experiences was associated with increased negative emotions, in support of previous research which has demonstrated similar responses to negative peer experiences (Goodman & Southam‐Gerow, 2010; Hubbard, 2001; Morrow et al., 2014; Reavis et al., 2015; Rose & Rudolph, 2006). Furthermore, peer nominations of emotional dysregulation were related to higher levels of peer‐reported physical and relational aggression, also consistent with prior findings (Bowie, 2010; Ersan, 2020; Garofalo et al., 2021; Kokkinos et al., 2019; Sullivan et al., 2010).

In line with previous findings, we also observed common gender differences in types of aggression, such that boys received more nominations for physical aggression than girls, and girls received more nominations for relational aggression than boys. Interestingly, boys also received more nominations for emotional dysregulation, which partially explained their higher rates of peer‐reported physical aggression. Thus, emotional dysregulation may play a role in physically aggressive externalizing behaviors that are unique for boys in early adolescence.

In full support of Hypothesis #4 and conditional support of Hypothesis #5, the effect of emotional dysregulation on physical aggression was partially through the indirect pathway of emotional reactivity, but only among boys. Although gender differences in emotional reactivity itself were not observed, the gender‐specific pathway may be related to the distinct emotional responses boys have evidenced in response to emotion‐inducing stimuli (Knight et al., 2002) or to the experience and regulation of anger specifically (e.g., Berkowitz, 1990; Eisenberg et al., 2001; Sullivan et al., 2010), both of which have been proposed as processes underlying gender differences in the expression of aggression. Furthermore, the absence of some associations among girls suggests that emotional reactivity may not operate in the same manner across genders. One possible explanation is that girls may employ different strategies for managing negative peer experiences, including relational coping or indirect expressions of distress, rather than overt aggression. In addition, relational aggression among girls may be more socially normative within peer contexts and therefore less dependent on emotional reactivity. These findings underscore the importance of considering gender‐specific emotional and interpersonal processes when examining aggression during adolescence. In addition, the predominantly middle‐class, Western cultural context of the sample may have shaped both emotional expression and peers' interpretations of aggression. For instance, Western cultural norms often place greater acceptance on emotional expressiveness and individual emotional experiences relative to more collectivistic cultural contexts that emphasize emotional restraint and interpersonal harmony. Such cultural norms may influence not only how adolescents experience and regulate emotions, but also how peers perceive and nominate aggressive or emotionally dysregulated behaviors. Similarly, the school context and peer‐group dynamics within predominantly European‐Canadian classrooms may have contributed to gender differences in patterns observed in the present study.

### Implications and strengths

Our study capitalized on intensive longitudinal data to develop an understanding of physical and relational aggression, a feature of several externalizing psychopathologies. In addition, to minimize recall biases and enhance ecological validity, these data provide an opportunity to understand the connection between daily experiences, emotional responses, and externalizing behavior in a way that neither cross‐sectional nor longitudinal research with longer measurement intervals can accomplish. With five assessments across the school day (over four days), the high‐temporal‐resolution design enabled a rich characterization of early adolescents' daily social experiences and emotional responses, thereby allowing accurate exploration of the processes underlying aggression. Another key contribution of the current study is the latent multilevel structural equation approach adopted herein. A number of studies have explored each component of the proposed model (see Figure 1). However, to our knowledge, the current study is the first to examine them all simultaneously. This analytic strategy allowed us to account for each of the principal associations above and beyond any of the others. In addition, this study demonstrates the benefits of a multi‐method approach integrating experience sampling, self‐report, and peer‐nomination data. Because peer nominations are generally more sensitive to salient, pronounced, or ongoing behaviors, they can reflect how emotional dysregulation and physical aggression in response to negative peer experiences are recognized in the social context as abnormal patterns of behavior, reinforcing the clinical significance of the findings.

Furthermore, the importance of emotional reactivity and emotional dysregulation as potential pathways to physical aggression among boys suggests that interventions targeting emotional awareness, emotion regulation, and adaptive responses to peer stress may be particularly beneficial during early adolescence. Because boys in the present study appeared more likely to respond aggressively following heightened negative emotional responses to peer experiences, helping adolescents recognize and regulate emotional responses in stressful interpersonal situations may reduce the likelihood that negative peer interactions escalate into aggressive behavior. School‐based programs that promote emotional competence, coping strategies, peer conflict management, and self‐regulatory skills may therefore represent promising avenues for reducing externalizing behaviors during adolescence. In addition, interventions such as mindfulness‐based approaches may be useful insofar as they strengthen emotional awareness and reduce impulsive and aggressive responding to stressful experiences (Gillions et al., 2019; Tao et al., 2021). In fact, a recent meta‐analysis indicated that mindfulness‐based intervention (MBI) may have long‐lasting effects (at follow‐ups of 1–4 years) and be particularly effective for children and adolescents with clinically significant levels of aggressive behavior (Tao et al., 2021). These effects may partially reflect the strategic design of such interventions, which explicitly target behavior change. In addition, mindfulness involves intentionally directing attention toward present experiences with openness and acceptance, at the same time minimizing excessive emotional or cognitive reactivity, therefore fostering healthier emotional processes (Wupperman et al., 2012). Moreover, mindfulness facilitates decentering, which refers to the ability to mentally step back from automatic thoughts and emotional reactions, allowing individuals to respond to stressful situations in a more flexible and adaptive manner (Wupperman et al., 2012).

There may also be other targets for intervention that would be advisable complements due to the reputational nature of adolescent social dynamics. Specifically, the use of peer report measures of aggression and emotional dysregulation corroborated that these constructs are elements of behavior salient to peer perception, and like other aspects of peer reputation, they may linger within the peer group even when the actual behavior may have been reduced (Prinstein et al., 2009). This is especially important given that peer group behaviors have been shown to predict and reinforce aggressive behavior (Erath et al., 2009). In this respect, the findings of this study also provide justification for interventions targeting the peer system surrounding aggressive individuals, or that proactively work to instill new prosocial patterns of behavior that may enhance social reputations. In fact, reinforcing positive peer experiences may be even more effective than simply reducing negative social dynamics (Suldo et al., 2015).

### Limitations and future directions

As with any research effort, the present study is subject to several limitations that should be considered when interpreting the findings. Most importantly, the present study relied on a secondary analysis of an existing longitudinal dataset, which introduces the possibility of what has been referred to as “original error” (Oyinlade, 1987), indicating that some limitations inherent in the original data collection process may transfer to subsequent analyses without direct control from the current researchers. Consequently, although the present analyses were conducted rigorously, the findings remain dependent on the structure and design of the original dataset. For example, in this study, emotional reactivity and both forms of aggression were assessed concurrently. As a result, the temporal precedence necessary to establish true mediation could not be determined. As such, the observed indirect effect should be interpreted as preliminary and with caution, rather than definitive evidence of mediation.

Moreover, it is important to acknowledge that the use of peer nominations for socially negative behaviors among younger adolescents may need further discussion. Although peer nominations have been commonly used in developmental research because peers are often well‐positioned to observe socially salient behaviors such as aggression and emotional dysregulation, asking participants to identify classmates who fit negative behavioral descriptions may have introduced some discomfort or perceived social pressure. This concern may be particularly relevant during early adolescence, when peer relationships and social reputation are important. To minimize these concerns, nominations were completed confidentially and independently. Nevertheless, alternative informants, such as teacher reports, may represent a less intrusive approach for assessing aggressive or dysregulated behavior in younger samples. At the same time, teacher reports may be less sensitive to peer‐group dynamics and behaviors that occur outside direct adult supervision. Therefore, future research may benefit from integrating multiple informants to resolve this imbalance. Furthermore, although peer nominations were collected within classrooms, classroom‐level clustering was not modeled due to the small number of participating classes (i.e., only five classrooms). Nevertheless, classroom climate may influence peer experiences and aggression, and future research should consider potential classroom‐level variability.

Furthermore, the present study focused exclusively on negative emotional responses and did not assess positive affective reactivity. Because participants completed negative emotion items across a range of daily experiences, including neutral and positive peer interactions, the operationalization of emotional reactivity in the present study was restricted to negative emotional responding. This focus may have reduced variability in emotional responses across some contexts and limits conclusions regarding broader emotional functioning. Future research would benefit from incorporating both positive and negative affective processes to provide a more comprehensive understanding of adolescent emotional reactivity.

Another limitation of this study that needs addressing is that there was no distinction made between proactive aggression (i.e., “cold,” premeditated and goal‐oriented; Bushman & Anderson, 2001) and reactive aggression (i.e., “hot,” impulsive and driven by anger; Bushman & Anderson, 2001), despite previous research indicating that there are differences in their emotional pathways. For example, reactive aggression has typically been found to be more related to negative emotionality (Moore et al., 2019) and emotion dysregulation (Preston & Anestis, 2020). However, proactive aggression may also have a distinct emotion profile characterized by low emotionality or low emotional reactivity (Moore et al., 2019; Preston & Anestis, 2020). Although we did not distinguish between the two (i.e., proactive vs. reactive aggression), this decision was justified by the notion that this dichotomy is outdated and may impede advances in aggression research because it neglects multi‐motivated aggressive acts and also “is confounded by the automatic‐controlled information‐processing dichotomy” (Bushman & Anderson, 2001; Roberton et al., 2012).

In addition, we did not explore other elements of emotional experience (e.g., emotional inertia or variability) and social information processing (e.g., attributions for social events) that may be relevant to emotion‐aggression pathways. Although previous research has already implicated these as important individual difference factors underlying aggressive tendencies (e.g., Crick & Dodge, 1996; Slaughter et al., 2020), little is known about how these factors impact behavior in real‐time social situations. Furthermore, the present study focused exclusively on negative emotional reactivity in response to adverse peer experiences. Although this focus was theoretically consistent with the study of aggression, it limits the scope of the conclusions because positive emotional experiences and adaptive emotional processes were not examined. Future research would benefit from investigating both positive and negative emotional reactivity to better understand the broader emotional dynamics underlying adolescent social behaviors.

Finally, emotional processes and how individuals express emotions may vary across cultural contexts (von Salisch et al., 2022), and further research is needed to examine the generalizability of these pathways. To elaborate, the relevance of cultural context should be considered when interpreting the present findings. The sample in this study consisted primarily of middle‐class, predominantly European‐Canadian adolescents. However, some participants reported a first language other than English. Even though all participants were fluent in English, the findings should be interpreted within the cultural and demographic context of this specific sample and may not generalize to the broader culturally and ethnically diverse Canadian adolescent population. For instance, Western and Eastern cultures differ in the extent to which high‐ versus low‐arousal emotions are experienced, preferred, and valued (Lim, 2016). Such differences indicate that the emotional processes underlying experience, expression, and social behavior are culturally shaped. Likewise, the manifestation of aggression also varies across cultures (Severance et al., 2013). For example, in many Asian cultural contexts, emotions are viewed as interpersonal in nature and are expected to be regulated and controlled to preserve social harmony (Chen et al., 2006). Consequently, Korean and Chinese children display less intense negative emotions in comparison to those in Western cultures (Lee et al., 2017). Yet, it remains unclear whether the real‐time processes driving these forms of aggression are universal or culturally specific. Future research should further investigate whether the moment‐to‐moment emotional processes linked to aggression operate similarly across cultural contexts.

## CONCLUSION

Despite some limitations, the present study offers several noteworthy contributions to the literature. First, it highlights the nuanced ways in which emotional processes contribute to adolescent aggression, underscoring the dynamic interplay between emotional dysregulation, emotional reactivity, and both physical and relational aggression. Second, using the Experience Sampling Method and collecting real‐time, intensive longitudinal data enables the detection of time‐sequenced patterns that reveal how intraindividual characteristics such as emotional dysregulation interact with immediate contextual factors to shape aggressive behavior as it unfolds in adolescents' daily lives. Finally, the use of a multi‐informant approach (i.e., combining peer nominations with self‐reports) enhanced the validity of the findings. Together, these methodological strengths position this study to advance understanding of adolescent aggression and provide a richer empirical foundation for developing targeted, context‐sensitive interventions.

## AUTHOR CONTRIBUTIONS

**Shaghayegh Sheri McVay:** Conceptualization; investigation; funding acquisition; writing – original draft; methodology; writing – review and editing; formal analysis; visualization; validation; software; project administration. **Jonathan Bruce Santo:** Conceptualization; investigation; writing – original draft; writing – review and editing; visualization; validation; methodology; software; formal analysis; project administration; supervision. **Ryan E. Adams:** Data curation; project administration; methodology; validation; writing – review and editing; investigation; resources. **William M. Bukowski:** Resources; supervision; data curation; project administration; methodology; validation; writing – review and editing; investigation; conceptualization.

## FUNDING INFORMATION

This material is based on work supported by the National Science Foundation Graduate Research Fellowship Program under Grant Number 2024375659, 2024, awarded to the first author. Any opinions, findings, and conclusions or recommendations expressed in this material are those of the authors and do not necessarily reflect the views of the National Science Foundation.

## CONFLICT OF INTEREST STATEMENT

The authors declare that no actual or perceived conflict of interest existed in the conduct and reporting of the research.

## ETHICS STATEMENT

Ethics approval statement is not applicable. The study involved a secondary analysis of anonymized data.

## CONSENT STATEMENT

Parent/guardian consent was obtained. In addition, all adolescents provided assent.

## Data Availability

The data that support the findings of this study are available from the corresponding author upon reasonable request.
